# Comparative Value of Echocardiography vs. Right Heart Catheterization in Heart Failure With Preserved Ejection Fraction

**DOI:** 10.1111/echo.70362

**Published:** 2025-12-05

**Authors:** Nathanael Tran, Mohammad Shaar, Hussein Al‐Sudani, Ramy Sedhom, Hamza Akhtar, Kevin Bryan Lo, Gregg S. Pressman

**Affiliations:** ^1^ Lahey Hospital & Medical Center Burlington Massachusetts USA; ^2^ Baptist Hospitals of Southeast Texas Beaumont Texas USA; ^3^ Jefferson Einstein Medical Center Thomas Jefferson University Philadelphia Pennsylvania USA; ^4^ Loma Linda University Loma Linda California USA; ^5^ Brigham and Women's Hospital Boston Massachusetts USA

**Keywords:** heart failure with preserved ejection fraction, pulmonary hypertension, right atrium, right heart catheterization, speckle‐tracking, strain imaging

## Abstract

**Background:**

Echocardiography and right heart catheterization (RHC) are useful in the evaluation of heart failure with preserved ejection fraction (HFpEF). We compared the two among decompensated HFpEF patients to determine their relative value in predicting all‐cause mortality and a composite of cardiovascular (CV) death and heart failure (HF) readmission at 1 year.

**Methods:**

One hundred seventy‐eight decompensated HFpEF patients who underwent both procedures and survived to hospital discharge were retrospectively identified. Hospital records and the National Death Index were queried to determine outcomes. Echocardiographic and invasive parameters were tested for significant associations with each outcome. Additionally, three predictive models were developed: Model‐1: basic demographics and comorbidities, Model‐2: adding echocardiographic parameters, and Model‐3: adding invasive hemodynamic parameters.

**Results:**

For mortality (*n* = 26; 15%), echocardiographic *E*/average *e′*, invasive mean pulmonary artery pressure (mPAP), and pulmonary capillary wedge pressure (PCWP) showed significant associations. Model‐1 yielded a *c*‐statistic of 0.65; 95% CI (0.54–0.76). Adding echocardiographic and hemodynamic variables produced a nonsignificant increase to 0.71; 95% CI (0.60–0.82), though mPAP remained an independent predictor (OR 1.05; 95% CI 1.01–1.09, *p* = 0.025). For the composite outcome (*n* = 31; 17%), significant associations were found for echocardiographic right atrial reservoir strain (RAsr), *E*/average *e′*, and invasive right atrial pressure, mPAP, and PCWP. Model‐1 had a *c*‐statistic of 0.70; 95% CI (0.60–0.79). Adding echocardiographic parameters significantly increased this to 0.81; 95% CI (0.73–0.88). Adding hemodynamic parameters (model‐3) resulted in a nonsignificant increase to 0.83; 95% CI (0.76–0.90).

**Conclusions:**

Among decompensated HFpEF patients, the addition of echocardiographic parameters significantly improved model prediction for the composite endpoint of CV death and HF hospitalization. Further addition of invasive hemodynamic variables did not.

## Graphical abstract

Adding echocardiographic parameters (right atrial reservoir strain [RAsr] and *E*/*e′*) to a basic model of demographics and comorbidities significantly improved discrimination of cardiovascular (CV) outcomes (area under the ROC curve) among decompensated HFpEF patients. The addition of right heart catheterization (RHC) parameters, by contrast, did not significantly improve the area under the ROC curve.

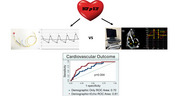

**Abbreviations**
AHA: American Heart Association
ASE: American Society of Echocardiography
BNP: brain natriuretic peptide
CV: cardiovascular
EACVI: European Association of Cardiovascular Imaging
HF: heart failure
HFpEF: heart failure with preserved ejection fraction
HFrEF: heart failure with reduced ejection fraction
LV: left ventricular
mPAP: mean pulmonary artery pressure
PADP: pulmonary artery diastolic pressure
PASP: pulmonary artery systolic pressure
PCWP: pulmonary capillary wedge pressure
PH: pulmonary hypertension
RA: right atrial
RAsr: right atrial reservoir strain
RHC: right heart catheterization
RVSP: right ventricular systolic pressure
STE: speckle‐tracking echocardiography

## 1 Introduction

Heart failure (HF) is a complex clinical syndrome that is a frequent cause of morbidity and mortality. Approximately half of patients with clinical HF have a preserved ejection fraction (HFpEF) and represent a challenging population to manage [1]. Patients with HFpEF are prone to developing pulmonary hypertension (PH), either from increased left ventricular (LV) filling pressure and/or intrinsic pulmonary vascular abnormalities. These patients have a worse overall prognosis and often present with right ventricular dysfunction [2, 3, 4, 5]. Although hemodynamic assessment with right heart catheterization (RHC) is the gold standard for evaluation of HFpEF and PH, this is not done routinely due to its invasive nature, complexity, and cost considerations [6]. Instead, it is generally reserved for cases where diagnosis remains uncertain after noninvasive testing, or in acute decompensation where there is possible diuretic resistance, concern for low cardiac output, or equivocal volume status on a background of worsening kidney function [1, 6, 7].

Echocardiography is widely employed in the assessment of HF patients, and there is growing interest in the use of myocardial strain via speckle‐tracking techniques (STE). When applied to the atria, strain reflects chamber compliance and myocardial contractility [8]. Both left and right atrial strain have been shown to be abnormal in patients with HFpEF compared to healthy controls [9]. Left atrial strain has also been shown to correlate with clinical outcomes in HF patients [10, 11], and there is increasing evidence that right atrial (RA) strain is predictive of cardiovascular (CV) endpoints in patients with HFpEF [5, 12, 13]. Contemporary evidence also notes the prognostic utility of RA strain in patients with nonischemic cardiomyopathy [14].

Methods such as strain imaging for assessment of decompensated HF remain underutilized and may provide useful prognostic information, particularly when RHC is not immediately available. However, data comparing both invasive and noninvasive modalities with clinical outcomes in this patient population remain scarce. We therefore sought to evaluate the prognostic ability of echocardiography, including atrial and ventricular strain, as compared to invasive RHC in patients with decompensated HFpEF.

## 2 Methods

This was a retrospective cross‐sectional study which included patients hospitalized for acute HF exacerbation. Hospital records were searched for the period between January 2010 to December 2016 to identify patients with a primary discharge diagnosis of “Heart Failure with Preserved Ejection Fraction (HFpEF)” or “Diastolic Heart Failure”. HFpEF was defined as an ejection fraction of 50% or greater along with signs and symptoms of HF, elevated brain natriuretic peptide (BNP), or evidence of diastolic dysfunction as confirmed by review of echocardiogram reports. Of this pool of potential subjects, only those who had RHC during the index admission were eligible for inclusion. We excluded patients with end‐stage renal disease on hemodialysis, acute myocardial infarction, severe valvular disorders, complex congenital heart disease, or severe chronic obstructive pulmonary disease, as well as those who died during the index hospitalization.

**FIGURE 1:**
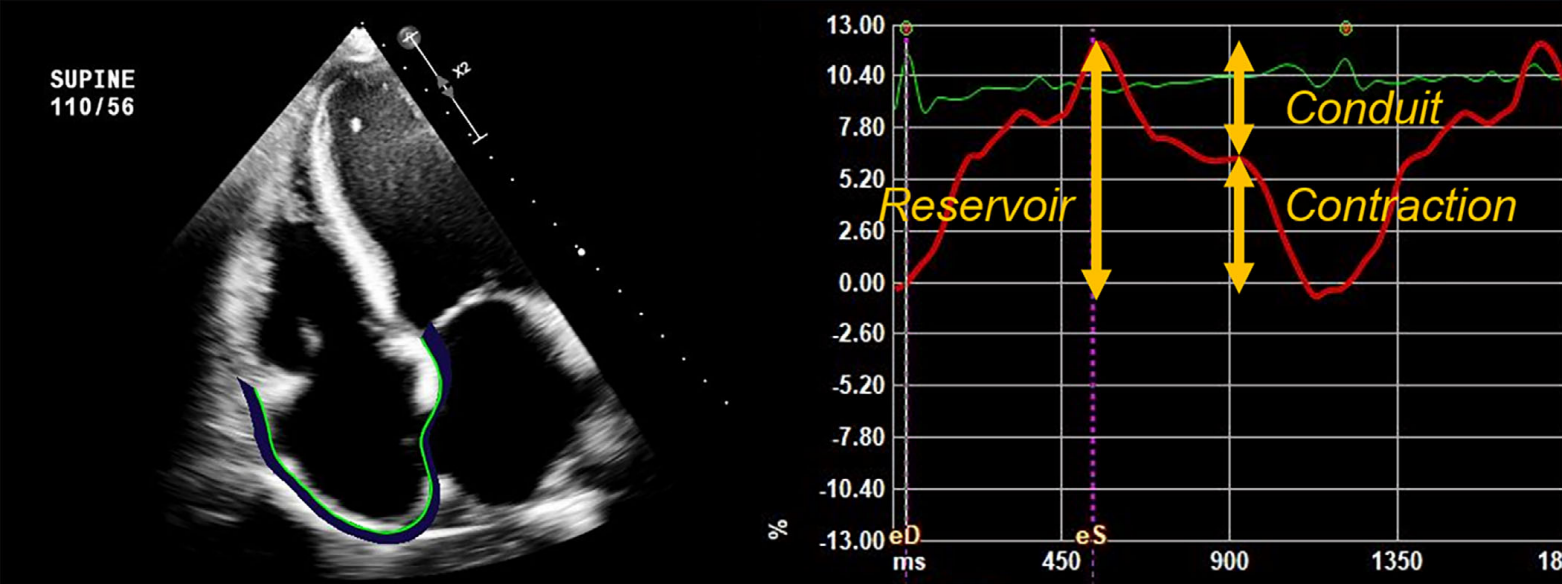
Sample right atrial strain curve demonstrating measurement of reservoir, conduit, and contraction strain.

All subjects underwent Doppler echocardiography according to American Society of Echocardiography (ASE)/European Association of Cardiovascular Imaging (EACVI) standards. LV diastolic diameter was assessed using two‐dimensional‐guided linear measurements in the parasternal long‐axis view. LV ejection fraction was calculated using the modified Simpson's Biplane method of disks. Left atrial volume was calculated using the biplane disk summation technique and indexed to body surface area. RA volume was calculated using the single‐plane disk summation technique in a dedicated apical four‐chamber view and indexed to body surface area. Diastolic dysfunction was assessed according to the 2016 ASE/EACVI update to the guidelines for evaluation of diastolic function. Two‐dimensional strain analysis was performed using vendor‐neutral software (TomTec Imaging Systems, Germany). For the right ventricle, the apical four‐chamber view was utilized; three landmark points were set, and strain curves of the basal, middle, and apical segments of the right ventricular free wall and septum were generated. Right ventricular free wall strain and fractional area change were automatically calculated. RA reservoir, conduit, and contraction strain were obtained with a zero‐strain reference set at end diastole (Figure 1). Strain curves were accepted only after visual assessment confirmed good tracking of the cardiac borders. Similar methods were used for the left ventricle and left atrium using the preset parameters built into the software. Demographic and clinical parameters, along with RHC hemodynamic measurements, were abstracted from the electronic medical record. Clinical parameters included are a history of hypertension, diabetes mellitus, atrial fibrillation, coronary artery disease, chronic kidney disease, and BNP level (pg/mL).

The outcomes of interest were as follows: (1) all‐cause mortality and (2) a composite of CV death and HF readmission in the year following admission. CV death was defined in accordance with the 2017 American Heart Association (AHA) consensus report established for clinical trials as sudden cardiac death, death due to acute myocardial infarction or HF, death due to ischemic stroke, CV procedures, or CV hemorrhage, or death due to other CV causes [15]. Mortality data were obtained via a chart review query of the National Death Index. This study was approved by the Institutional Review Board of the Einstein Healthcare Network.

### 2.1 Statistical Analysis

Data are presented and stratified according to the presence or absence of the mortality outcome (all‐cause death at 1 year) and the composite outcome (CV death+HF readmission at 1 year) (please see summary Tables S1 and S2). Categorical demographic and clinical variables are presented using frequencies and percentages and were compared using the chi square test. Continuous variables that were normally distributed are presented using means±standard deviations with comparisons made using independent *T* tests; for skewed variables medians and interquartile range are presented with the Wilcoxon rank sum test used for comparisons. Three models were created for further analysis: model 1 consisting of demographic parameters and comorbidities, model 2 representing the addition of significant echocardiographic variables to model 1, and model 3 representing addition of significant hemodynamic variables (from RHC) to model 2. Multivariable logistic regression analysis was then done for each of the endpoints and each of the models. Variables with *p* values < 0.2 were selected and included in the models. Predictors were checked for collinearity and removed from the model if > 0.70 correlated with any other variable. As a result, only 1 type of atrial strain (reservoir strain) was included in the models as it is less limited by the presence of atrial fibrillation. Collinear hemodynamic variables were also omitted. In particular pulmonary capillary wedge pressure (PCWP) was removed as it was collinear with mean pulmonary artery pressure (mPAP). Goodness of fit testing was done for each individual model. Results are presented using odds ratios and 95% confidence intervals. ROC analysis was performed to generate area under the curve (AUC, a.k.a. *c*‐statistic) values to estimate the predictive performance of each individual model. Comparison between different models and AUCs were done using the chi square test algorithm by DeLong, DeLong, and Clarke‐Pearson [16]. A *p* value of < 0.05 was considered statistically significant. All analyses were done using Stata Version 17 (Statacorp, College Station, TX).

## 3 Results

A total sample of 200 consecutive patients with complete RHC data and available echocardiographic data was identified. Those who did not survive to hospital discharge were excluded leaving a final sample of 178 patients. Mean age was 69.4 ± 13.5 years. Half were African American (50%) and 62% were female. Diabetes was present in 53% while 88% had hypertension. Median BNP was 514 (IQR 240–1261 pg/mL). See summary Tables 1, 2, 3, 4 for a full listing of clinical, echocardiographic, and hemodynamic parameters stratified by outcomes. Reasons for RHC were evaluation of volume status (78%) or suspected PH (12%).

**TABLE 1:** Summary table of clinical, echocardiographic, and hemodynamic variables according to 1‐year all‐cause mortality.

	Died (*n* = 26)	Survived (*n* = 152)	*p* value
Age	73.1 ± 10.8	68.8 ± 13.9	0.16
Male gender	12 (46%)	56 (37%)	0.37
BMI	33.3 ± 8.2	32.5 ± 8.0	0.49
Race			0.18
African American	12 (46%)	77 (51%)	
Caucasian	14 (54%)	61 (40%)	
Others	0 (0%)	14 (9%)	
HTN	22 (85%)	134 (88%)	0.61
DM	16 (62%)	79 (52%)	0.37
Atrial fibrillation	9 (35%)	37 (24%)	0.27
CAD	16 (62%)	72 (47%)	0.18
CKD	11 (42%)	41 (27%)	0.11
BNP (pg/mL)	394 (256–684)	538 (239–1355)	0.21
**Echocardiographic parameters**: Median (IQR)			
LA conduit strain (%)	12.4 (9.3–17.6)	13.7 (8.6–20.5)	0.48
LA contraction strain (%)	13.6 (8.5–20.1)	10.3 (6.2–17.7)	0.30
LA reservoir strain (%)	27.7 (19.7–33.3)	27.5 (17.5–37.4)	0.99
LVGLS (%)	15.1 (12.7–17.1)	15.5 (12.0–8.0)	0.90
LA volume(index) (mL/m^2^)	51.8 (44.7–63.0)	48.9 (36.7–66.0)	0.48
RA conduit (%)	10.4 (6.5–20.3)	11.5 (6.5–18.6)	0.97
RA reservoir strain (%)	29.4 (17.4–34.1)	24.8 (17.0–33.0)	0.43
RA contraction strain (%)	12.7 (7.4–20.0)	11.2 (6.5–17.7)	0.39
RV free wall strain (%)	15.4 (10.0–22.1)	15.1 (10.5–20.7)	0.93
RV FAC (%)	30.0 (15.0–35.0)	27.1 (17.8–39.4)	0.80
RA volume(index) (mL/m^2^)	30.0 (20.4–36.2)	24.7 (17.1–33.1)	0.25
*E*/*A* ratio	1.1 (0.8–1.3)	1.1 (0.8–1.5)	0.48
*E*/average *e′*	***19.7 (12.7*–*24.2)***	***14.3 (10.8*–*18.9)***	***0.02***
**Hemodynamic parameters**: Median (IQR)			
RA (mmHg)	13 (9–18)	11 (8–16)	0.30
RV systolic (mmHg)	***56 (45*–*65)***	***48 (40*–*60)***	***0.04***
RV diastolic (mmHg)	10 (7–20)	10 (5–15)	0.23
mPAP (mmHg)	***38 (33*–*45)***	***30 (25*–*40)***	***0.008***
PA systolic (mmHg)	***57 (50*–*65)***	***47 (39*–*60)***	***0.02***
PA diastolic (mmHg)	26 (18–32)	20 (16–27)	0.06
PVR (Dyn s/cm⁵)	240 (147–379)	206 (116–320)	0.35
PCWP (mmHg)	***25 (16*–*26)***	***19 (13*–*25)***	***0.04***
Fick‐CO (L/min)	5.1 (4.1–6.2)	4.7 (3.8–5.9)	0.39
CI (L/min/m^2^)	2.4 (2.0–2.8)	2.3 (2.0–2.8)	0.88
MAP (mmHg)	93 (83–110)	94 (86–106)	0.83
SVR (Dyn s/cm⁵)	1385 (1011–1565)	1371 (1097–1766)	0.46

*Note*: Bold italics indicate statistical significance.

Abbreviations: BMI, body mass index; BNP, brain natriuretic peptide; CAD, coronary artery disease; CI, cardiac index; CKD, chronic kidney disease; DM, diabetes mellitus; FAC, fractional area change; Fick‐CO, Fick‐cardiac output; HTN, hypertension; LA, left atrial; LVGLS, left ventricular global longitudinal strain; MAP, mean arterial pressure; mPAP, mean pulmonary artery pressure; PA, pulmonary artery; PCWP, pulmonary artery capillary wedge pressure; PVR, pulmonary vascular resistance; RA, right atrial; RV, right ventricular; SVR, systemic vascular resistance.

**TABLE 2:** Summary table of clinical, echocardiographic, and hemodynamic variables according to 1‐year composite outcome.

	Composite outcome (*n* = 31)	Without outcome (*n* = 147)	*p* value
Age	70.6 ± 14.2	69.2 ± 13.4	0.61
Male gender	14 (45%)	54 (37%)	0.38
BMI	34.1 ± 9.8	32.3 ± 7.6	0.25
Race			0.55
African American	17 (55%)	72 (49%)	
Caucasian	13 (42%)	62 (42%)	
Others	1 (3%)	13 (9%)	
HTN	27 (87%)	129 (88%)	0.91
DM	20 (65%)	75 (51%)	0.17
Atrial fibrillation	10 (32%)	36 (24%)	0.37
CAD	***21 (68%)***	***67 (46%)***	***0.03***
CKD	***14 (45%)***	***38 (26%)***	***0.03***
BNP (pg/mL)	421 (181–1066)	519 (251–1266)	0.37
**Echocardiographic parameters**: Median (IQR)			
LA conduit strain (%)	11 (7–17)	14 (9–21)	0.07
LA contraction strain (%)	10 (5–19)	11 (7–18)	0.65
LA reservoir strain (%)	22 (16–32)	28 (18–37)	0.12
LVGLS (%)	16 (11–18)	15 (12–18)	0.73
LA volume(index) (mL/m^2^)	53 (44–62)	49 (37–66)	0.29
RA conduit (%)	***8 (5*–*12)***	***12 (7*–*19)***	***0.01***
RA reservoir strain (%)	***20 (11*–*30)***	***26 (18*–*34)***	***0.01***
RA contraction strain (%)	8 (6–15)	12 (8–19)	0.08
RV free wall strain (%)	14 (9–20)	15 (11–21)	0.30
RV FAC (%)	29 (14–40)	28 (19–39)	0.94
RA volume(index) (mL/m^2^)	29 (21–33)	24 (17–34)	0.48
*E*/*A* ratio	1.2 (0.8–1.5)	1.1 (0.8–1.5)	0.56
*E*/average *e′*	***19.8 (13.5*–*26.2)***	***14.2 (10.7*–*19.2)***	***0.003***
**Hemodynamic parameters**: Median (IQR)			
RA (mmHg)	15 (8–19)	10 (8–16)	0.07
RV systolic (mmHg)	***60 (45*–*68)***	***48 (40*–*60)***	***0.004***
RV diastolic (mmHg)	12 (7–20)	10 (5–15)	0.11
mPAP (mmHg)	***36 (30*–*46)***	***30 (24*–*40)***	***0.007***
PA systolic (mmHg)	***59 (50*–*68)***	***46 (39*–*60)***	***0.001***
PA diastolic (mmHg)	***25 (19*–*38)***	***20 (16*–*27)***	***0.01***
PVR (Dyn s/cm⁵)	264 (123–389)	208 (117–320)	0.50
PCWP (mmHg)	***25 (20*–*30)***	***18 (13*–*25)***	***0.002***
Fick‐CO (L/min)	4.7 (3.7–6.0)	4.7 (3.9–6.0)	0.76
CI (L/min/m^2^)	2.3 (1.8–2.8)	2.3 (2.0–2.9)	0.45
MAP (mmHg)	93 (83–103)	96 (86–107)	0.48
SVR (Dyn s/cm⁵)	1283 (1054–1643)	1423 (1097–1766)	0.28

*Note*: **Bold italics** indicate statistical significance.

Abbreviations: BMI, body mass index; BNP, brain natriuretic peptide; CAD, coronary artery disease; CI, cardiac index; CKD, chronic kidney disease; DM, diabetes mellitus; FAC, fractional area change; Fick‐CO, Fick‐cardiac output; HTN, hypertension; LA, left atrial; LVGLS, left ventricular global longitudinal strain; MAP, mean arterial pressure; mPAP, mean pulmonary artery pressure; PA, pulmonary artery; PCWP, pulmonary artery capillary wedge pressure; PVR, pulmonary vascular resistance; RA, right atrial; RV, right ventricular; SVR, systemic vascular resistance.

**TABLE 3:** Multivariable logistic regression models for 1‐year all‐cause mortality.

Variables	Model 1 demographics alone OR (95%CI)	Model 2 demographics + echo OR (95%CI)	Model 3 demographics + echo + hemodynamics OR (95%CI)
Age	1.02 (0.99–1.06)	1.02 (0.98–1.06)	1.03 (0.99–1.07)
Female	Reference	Reference	Reference
Male	1.51 (0.62–3.66)	1.23 (0.47–3.18)	1.48 (0.55–3.96)
Non‐African American	Reference	Reference	Reference
African American	1.02 (0.41–2.52)	0.81 (0.31–2.10)	0.67 (0.25–1.81)
CAD	1.52 (0.61–3.75)	1.50 (0.57–3.91)	1.50 (0.57–3.95)
CKD	1.93 (0.81–4.61)	2.17 (0.88–5.35)	1.87 (0.74–4.74)
*E*/average *e′*		1.02 (0.97–1.08)	1.01 (0.95–1.06)
mPAP			***1.05 (1.01*–*1.09)*** *
AUC (*c*‐statistic)	0.65 (0.54–0.76)	0.67 (0.55–0.79)	0.71 (0.60–0.82)

*Note*: Bold italics indicate statistical significance.

Abbreviations: AUC, area under the ROC curve; CAD, coronary artery disease; CKD, chronic kidney disease; mPAP, mean pulmonary artery pressure.

* *p* = 0.025.

**TABLE 4:** Multivariable logistic regression demographic and echo model for 1‐year composite outcome.

Variables	Model 1 demographics alone OR (95%CI)	Model 2 demographics + echo OR (95%CI)	Model 3 demographics + echo + hemodynamics OR (95%CI)
Age	1.00 (0.97–1.03)	1.00 (0.96–1.04)	1.00 (0.97–1.04)
Female	Reference	Reference	Reference
Male	1.66 (0.71–3.89)	1.36 (0.53–3.52)	1.52 (0.56–4.09)
Non‐African American	Reference	Reference	Reference
African American	1.49 (0.61–3.60)	2.04 (0.75–5.55)	1.82 (0.65–5.05)
DM	1.43 (0.60–3.39)	0.93 (0.36–2.40)	0.83 (0.31–2.22)
CAD	***2.70 (1.12*–*6.53)***	***3.60 (1.30*–*9.93)***	***3.82 (1.34*–*10.86)***
CKD	2.14 (0.93–4.94)	***3.63 (1.38*–*9.56)***	***3.43 (1.27*–*9.25)***
RA reservoir strain		***0.94 (0.90*–*0.98)***	***0.94 (0.90*–*0.98)***
*E*/average *e′*		***1.07 (1.02*–*1.12)***	***1.06 (1.01*–*1.12)***
RA pressure			1.05 (0.98–1.13)
mPAP			1.01 (0.96–1.06)
AUC (*c*‐statistic)	0.70 (0.60–0.79)	***0.81 (0.73*–*0.88)*** ^*^	0.83 (0.76–0.90)

*Note*: Bold italics *indicate statistical significance*.

Abbreviations: AUC, area under the ROC curve; CAD, coronary artery disease; CKD, chronic kidney disease; DM, diabetes mellitus; mPAP, mean pulmonary artery pressure; RA, right atrial.

* model 2 vs. model 1, *p* = 0.0039 and model 3 vs. model 2, *p* = 0.43.

**FIGURE 2:**
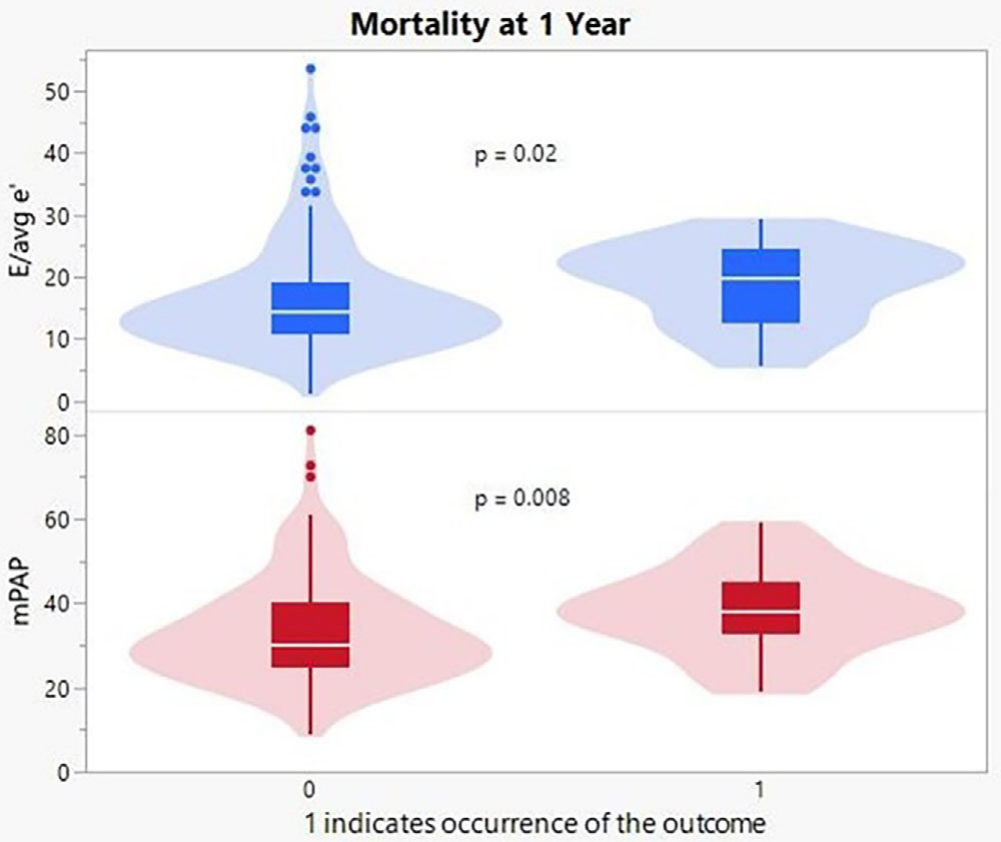
Violin/Box plots displaying higher *E*/avg *e′* (blue) and higher mPAP (red) in those experiencing the mortality outcome. mPAP, mean pulmonary artery pressure.

One‐year all‐cause mortality rate was 15% (*n* = 26; all deaths including non‐CV deaths) while the 1‐year composite outcome of CV death or HF readmission was 17% (*n* = 31; 10 CV deaths and 21 HF readmissions). Comparing patients with vs. without 1‐year all‐cause mortality there were no significant differences in demographics or comorbidities on univariate analysis (Table 1). For echo variables only *E*/average *e′* ratio was significantly different between groups. Among hemodynamic parameters right ventricular systolic pressure (RVSP), mPAP, pulmonary artery systolic pressure (PASP), and PCWP were significantly different between groups (Figure 2). RVSP and PASP were found to be collinear with mPAP as was PCWP; these variables were thus excluded from further analysis. The *c*‐statistic for model 1 (demographic parameters and comorbidities) was only 0.65 (95% CI 0.54–0.76) indicating poor ability to discriminate between those who died and those who survived. Adding *E*/average *e′* ratio to the analysis (model 2) did not significantly improve the *c*‐statistic (0.67; 95% CI 0.55–0.79). Further addition of mPAP (model 3) also did not significantly improve the *c*‐statistic (0.71; 95% CI 0.60–0.82) though mPAP remained significantly associated with 1‐year all cause‐mortality (OR 1.05 95% CI 1.01–1.09, *p* = 0.025). See Table 3 for full results.

**FIGURE 3:**
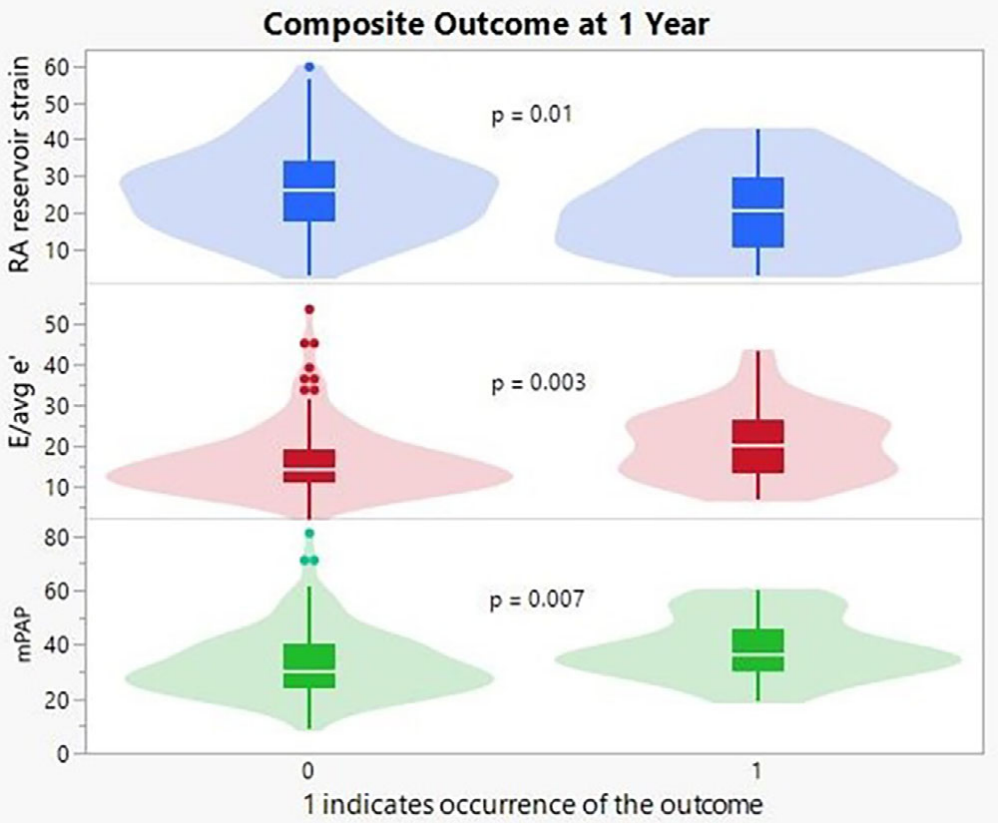
Violin/Box plots displaying the composite outcome of CV death and HF readmission at 1‐year being associated with lower RAsr (blue), higher *E*/avg *e′* (red), and higher mPAP (green). CV, cardiovascular; HF, heart failure; mPAP, mean pulmonary artery pressure; RA, right atrial.

**FIGURE 4:**
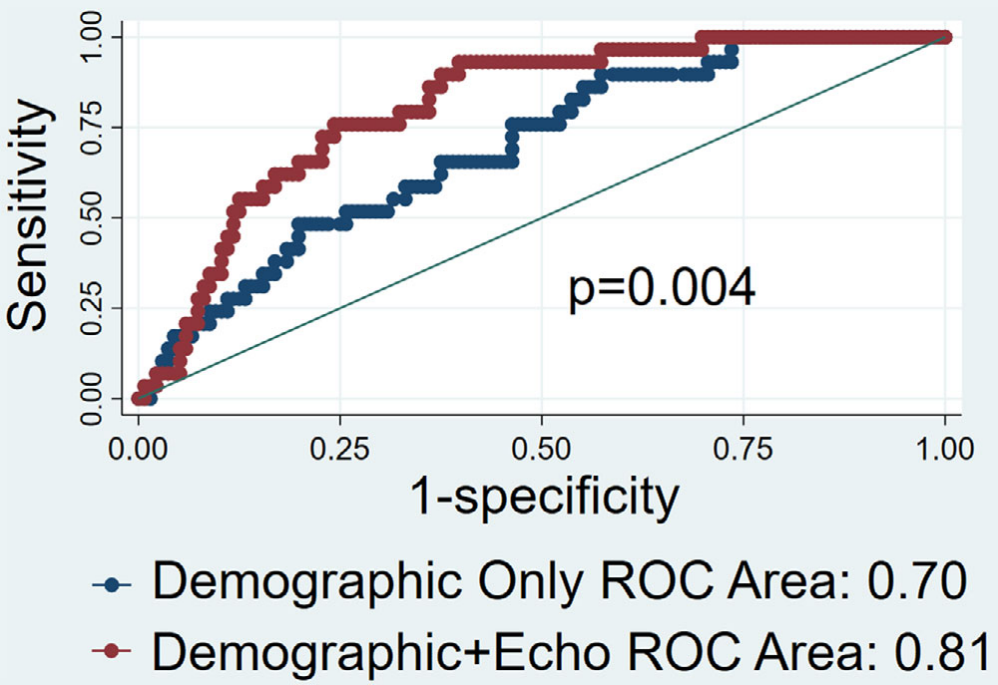
ROC curves predicting the composite outcome of CV death and HF readmissions at 1‐year comparing model 1 (clinical variables, blue) and model 2 (clinical + echo variables, red). The addition of echo variables significantly improved the area under the ROC (*c*‐statistic). CV, cardiovascular; HF, heart failure.

Looking at the composite outcome of CV death and HF readmission at 1 year, RA strain and *E*/average *e′* were the echocardiographic variables that were significantly different between groups on univariate analysis. Among the hemodynamic variables RA pressure, RVSP, mPAP, PASP, pulmonary artery diastolic pressure (PADP), and PCWP were significantly different between groups (Table 2, Figure 3). Because of collinearity RVSP, PASP, PADP, and PCWP were removed from further analysis. The *c*‐statistic for model 1 (demographic parameters and comorbidities) was 0.70 (95% CI 0.60–0.79) indicating poor ability to discriminate between those who experienced the outcome vs. those who did not. Adding echocardiographic variables RA reservoir strain (RAsr) and *E*/average *e′* ratio (model 2) increased the *c*‐statistic to 0.81 (95% CI 0.73–0.88), a statistically significant improvement (*p* = 0.004, Figure 4). Further addition of mPAP did not significantly change the *c*‐statistic (0.83; 95% CI 0.76–0.90) (Table 4).

## 4 Discussion

To our knowledge, this is the first study comparing the prognostic impact of echocardiographic strain variables with hemodynamic data in patients with acutely decompensated HFpEF. We found that the composite outcome of CV death/HF readmission could be discriminated reasonably well by adding the echocardiographic parameters RAsr and *E*/average *e′* to a basic demographics and comorbidities model. Further addition of hemodynamic parameters did not significantly improve model discrimination. Thus, echocardiographic parameters may be better predictors of the composite outcome than hemodynamic parameters obtained from RHC.

By contrast, our models were unable to effectively discriminate (i.e., low *c*‐statistic) all‐cause death at 1 year. The final model, incorporating *E*/average *e′* and mPAP, did not significantly improve on the low *c*‐statistic associated with the basic demographics and comorbidities model. In essence, neither echocardiographic nor hemodynamic variables, when added to a basic demographics and comorbidities model, were able to distinguish survivors from those experiencing all‐cause death.

HFpEF encompasses a heterogenous patient population with approximately 50% developing PH [3]. PH is thought to arise via increased LV filling pressure leading to a sustained increase in left atrial pressure. This in turn results in characteristic pathologic changes in the pulmonary vasculature that are more pronounced in HFpEF than heart failure with reduced ejection fraction (HFrEF) and are thought to involve inflammation [17]. The development of PH in HFpEF is a well‐established risk factor for poor outcome with a 5‐year mortality of approximately 50% [2]. In the current study mPAP, as obtained via RHC, was associated with all‐cause mortality. However, the strength of the association was not high, likely because patients with PH‐HFpEF have many comorbidities including obesity, metabolic syndrome, diabetes mellitus type 2, and renal dysfunction all of which can contribute to mortality. This may also explain why echocardiographic parameters generally were not associated with all‐cause mortality. The exception was *E*/average *e′* which showed a statistically significant, but again weak, association on univariate analysis. However, this did not remain significant on multivariate analysis. Applying the various models for prediction of all‐cause death, the basic demographics and comorbidities model showed poor association with the mortality endpoint. Adding *E*/average *e′* (model 2) and mPAP (model 3) resulted in nominal increases in the *c*‐statistic which were not statistically significant.

When looking at the composite endpoint of CV death and HF readmission, our data show remarkably different results. The echocardiographic parameters RAsr and *E*/average *e′* were found to be independently associated with the outcome at 1 year while mPAP was not. Adding these parameters to a basic demographics and comorbidities model increased the *c*‐statistic on ROC analysis from 0.70 to 0.81, a statistically significant result. This implies that the combined model correctly discriminated between subjects who experienced the outcome from those who did not 81% of the time vs. only 70% of the time for the basic model. Further adding hemodynamic variables did not significantly change this result.

Strain is a measure of myocardial deformation that was initially established as a less load‐dependent measure of LV contractility than ejection fraction. It has since been shown to have prognostic importance in aortic stenosis, even when the ejection fraction is normal [18]. LV global longitudinal strain also shows promise for early detection of chemotherapy‐induced cardiomyopathy [19, 20]. More recently, attention has turned to left atrial strain and its prognostic value. The three phases of atrial function can be characterized by STE: reservoir strain during atrial filling, conduit strain during passive emptying into the ventricle, and booster/contraction strain during atrial systole [8]. Left atrial strain has been shown to have utility in predicting LV filling pressure and has prognostic value in both HFrEF and HFpEF [21, 22].

In our study of patients with decompensated HFpEF, RAsr, and *E*/average *e′*, were independently associated with the composite outcome of CV death and HF readmission at 1 year. The right atrium plays an important role in the circulatory system via right ventricular filling, thus helping to ensure adequate cardiac output, and central venous pressure regulation, both of which are associated with clinical outcomes [23, 24]. Patients with HFpEF are subject to chronic volume overload that can result in RA remodeling (dilatation) and dysfunction (reduced strain) over time [13, 23]. The mechanisms driving RA remodeling and dysfunction are complex but generally reflect increased afterload (due to increased RV end‐diastolic pressure), increased preload (from tricuspid regurgitation), and effects of atrial fibrillation if present [23]. There is increasing interest in the clinical utility of RA strain. Previous work, using STE to measure strain, has found RA strain to have prognostic value in patients with pulmonary arterial hypertension [25, 26]. RA strain, as measured by cardiac MRI (CMR) was shown to be a predictor of death in a mixed HF population [12, 25, 26]. RAsr among patients with HFpEF has also been associated with lower cardiac output and right ventricular dysfunction during exercise [13]. Our findings now extend the usefulness of this measure to patients with decompensated HFpEF where it appears to have prognostic importance for CV death and HF readmission.

Other echocardiographic measures of myocardial function, such as LV global longitudinal strain, left atrial strain, and right ventricular strain, were not found to differ significantly between groups in this study. They were thus not useful in predicting either outcome. These measures generally have relevant prognostic value in a variety of cardiac diseases. In the current study, each was substantially below the lower limit of normal thus identifying our patients as having very abnormal physiology. This is consistent with their being sick enough to require RHC. It is thus even more interesting that RA strain was alone among strain measures in having prognostic value for the composite outcome.

Aside from RAsr, we also found *E*/average *e′* to be independently associated with the composite CV death/HF readmission outcome. This may appear intuitive, but it is important to bear in mind that, while this parameter provides an estimate of LV filling pressure, it does not only reflect LV filling pressure. Rather, it incorporates the velocity of the early diastolic filling wave (*E*) and a measure of global LV relaxation (*e′*). *E*‐wave velocity can be affected by atrial stiffness as well as the gradient between LA and LV [27]. Tissue Doppler e’ is relatively, but not entirely, independent of loading conditions. Thus, *E*/*e′* reflects both hemodynamic conditions and intrinsic properties of both the LV and left atrial myocardium.

From a practical viewpoint, RHC is useful in confirming a diagnosis of HFpEF and perhaps in guiding treatment. However, our study suggests it is not useful in assessing CV outcomes at 1 year. In addition, RHC is not universally available and is an invasive procedure. By contrast, echocardiography has widespread availability, is noninvasive, and is already performed in nearly all patients with HFpEF. Based on the current results, it appears to have prognostic utility in decompensated HFpEF. Having the ability to identify patients at higher risk of adverse outcomes could allow more appropriate counseling, closer follow‐up, and more effective use of targeted therapies.

The limitations of our study include its retrospective design, relatively small size, and selection bias toward a sicker cohort who required RHC. Ideally, both STE and RHC parameters should be compared prospectively in a larger number of acutely decompensated HFpEF patients. However, our data do provide strong evidence that RAsr and *E*/average *e′* are important factors in determining CV outcomes as validated by the National Death Index and documented HF readmissions. Another limitation of our study is the likely heterogeneity of the study sample. We did not apply strict criteria for determining the presence of HFpEF, but rather enrolled subjects based on the treating physician's diagnosis. On the other hand, this sample is likely reflective of “real world” patients who receive a diagnosis of HFpEF. Lastly, our patients required RHC as part of their acute inpatient management thus representing a sicker cohort of patients with HFpEF.

## 5 Conclusions

This study supports the utility of RA strain and *E*/average *e′* as noninvasive parameters associated with CV outcomes in patients who present with decompensated HFpEF. Hemodynamic parameters obtained by RHC did not provide additional prognostic information for this outcome.

## Funding

The authors have nothing to report.

## Conflicts of Interest

The authors declare no conflicts of interest.

## Supporting information




**Supplementary Table 1**: Multivariable logistic regression models for 1‐year all cause mortality excluding some of the non‐significant variables (sensitivity analysis).
**Supplementary Table 2**: Multivariable logistic regression models for 1‐year composite outcome excluding some of the non‐significant variables (sensitivity analysis).

## Data Availability

The data that support the findings of this study are available on request from the corresponding author. The data are not publicly available due to privacy or ethical restrictions.
