# Supplementary material for: Comparative Value of Echocardiography vs. Right Heart Catheterization in Heart Failure With Preserved Ejection Fraction
Source: Echocardiography. 2025 Dec 5;42(12):e70362. doi: 10.1111/echo.70362 (PMC12679061; doi:10.1111/echo.70362)
Supplement: Supplementary file 1 — Supplementary Table 1: Multivariable logistic regression models for 1‐year all cause mortality excluding some of the non‐significant variables (sensitivity analysis). Supplementary Table 2: Multivariable logistic regression models for 1‐year composite outcome excluding some of the non‐significant variables (sensitivity analysis). [file ECHO-42-e70362-s001.docx]

**Supplementary Table 1. Multivariable logistic regression models for 1-year all cause mortality excluding some of the non-significant variables (sensitivity analysis).**

| Variables | Model 1  Demographics alone  OR (95%CI) | Model 2 Demographics + echo  OR (95%CI) | Model 3 Demographics + echo + hemodynamics  OR (95%CI) |
| --- | --- | --- | --- |
| Age | 1.02 (0.98-1.06) | 1.02 (0.98-1.06) | 1.03 (0.99-1.07) |
| Non-African American | Reference | Reference | Reference |
| African American | 0.91 (0.38-2.18) | 0.81 (0.31-2.10) | 0.61 (0.23-1.60) |
| CAD | 1.51 (0.61-3.70) | 1.50 (0.57-3.91) | 1.49 (0.57-3.91) |
| CKD | 1.94 (0.82-4.63) | 2.17 (0.88-5.35) | 1.88 (0.74-4.77) |
| E/average e’ |  | 1.02 (0.97-1.08) | 1.00 (0.95-1.06) |
| mPAP |  |  | ***1.04 (1.00-1.09)**** |
| AUC (c-statistic) | 0.66 (0.55-0.78) | 0.67 (0.55-0.79) | 0.71 (0.60-0.82) |

*p = 0.032

***Bold italics*** indicate statistical significance.

*AUC, area under the ROC curve; CAD*, coronary artery disease; *CKD,* chronic kidney disease; *mPAP,* mean pulmonary artery pressure.

**Supplementary Table 2. Multivariable logistic regression models for 1-year composite outcome excluding some of the non-significant variables (sensitivity analysis).**

| Variables | Model 1 Demographics alone  OR (95%CI) | Model 2  Demographics + echo  OR (95%CI) | Model 3 Demographics + echo + hemodynamics  OR (95%CI) |
| --- | --- | --- | --- |
| DM | 1.49 (0.64-3.46) | 1.08 (0.43-2.72) | 0.91 (0.35-2.37) |
| CAD | ***2.51 (1.09-5.77)*** | ***3.02 (1.18-7.78)*** | ***3.46 (1.29-9.30)*** |
| CKD | 2.15 (0.94-4.94) | ***3.42 (1.33-8.83)*** | ***3.29 (1.24-8.73)*** |
| RA reservoir strain |  | ***0.94 (0.90-0.98)*** | ***0.94 (0.90-0.98)*** |
| E/average e’ |  | ***1.06 (1.01-1.11)*** | ***1.06 (1.01-1.11)*** |
| RA pressure |  |  | 1.06 (0.98-1.14) |
| mPAP |  |  | 1.01 (0.96-1.06) |
| AUC (c-statistic) | 0.67 (0.58-0.77) | ***0.80 (0.72-0.88)**** | 0.81 (0.75-0.88) |

*model 2 vs model 1 p=0.003, model 3 vs model 2 p=0.46

***Bold italics*** indicate statistical significance.

*AUC, area under the ROC curve; CAD*, coronary artery disease; *CKD,* chronic kidney disease; *DM*, diabetes mellitus; *mPAP,* mean pulmonary artery pressure; *RA,* right atrial.
